# An exosome‐based liquid biopsy signature for therapeutic response prediction in metastatic gastric cancer

**DOI:** 10.1002/ctm2.1629

**Published:** 2024-07-19

**Authors:** Keisuke Okuno, Shuichi Watanabe, Hoon Hur, Jeeyun Lee, Joon Oh Park, Masanori Tokunaga, Minoru Tanabe, Yusuke Kinugasa, Ajay Goel

**Affiliations:** ^1^ Department of Molecular Diagnostics and Experimental Therapeutics Beckman Research Institute of City of Hope Biomedical Research Center Monrovia California USA; ^2^ Department of Gastrointestinal Surgery Tokyo Medical and Dental University Tokyo Japan; ^3^ Department of Hepatobiliary and Pancreatic Surgery Tokyo Medical and Dental University Tokyo Japan; ^4^ Department of Surgery Ajou University School of Medicine Suwon South Korea; ^5^ Division of Hematology‐Oncology Department of Medicine Samsung Medical Center Seoul South Korea; ^6^ City of Hope Comprehensive Cancer Center Duarte California USA

Dear Editor,

Systemic chemotherapy based on fluorouracil or fluoropyrimidine + platinum is recommended as first‐line chemotherapy for patients with metastatic gastric cancer (mGC)^1^, ^2^; however, the efficacy of such treatment regimens remains inadequate, with a median progression‐free survival (PFS) of ∼6 months.^3^, ^4^, ^5^, ^6^, ^7^ In the present study, we evaluated the feasibility of utilising an exosomal microRNA (exo‐miRNA)‐based liquid biopsy assay that can facilitate response prediction to first‐line systemic chemotherapy in patients with mGC by undertaking a comprehensive genome wide biomarker discovery effort, followed by independent quantitative reverse‐transcription polymerase chain reaction (qRT‐PCR)‐based biomarker validation.

The overall study design is described in Figure S1. For the biomarker discovery, frozen surgical tissues (*n* = 14) and pre‐treatment plasma specimens (*n* = 11) from patients with stage IV gastric cancer (GC) enrolled at Ajou University Hospital, South Korea (tissue‐ and exosome‐based discovery cohort, respectively) were analysed. For the biomarker training, pre‐treatment plasma specimens from 23 patients with stage IV GC enrolled at Ajou University Hospital (clinical training cohort) were examined, and these patients were independent of the biomarker discovery cohort specimens. Pre‐treatment plasma specimens from 33 patients with stage IV GC enrolled at Samsung Medical Center, South Korea (clinical validation cohort) were examined for biomarker validation. The patient characteristics of each cohort are shown in Tables S1–S3. The first‐line chemotherapy regimens were based on fluorouracil, fluoropyrimidine and platinum, as Japanese GC treatment guidelines recommended.^2^ Due to these treatments’ median PFS of 6 months in previous large clinical trials, for the response definition in this study, patients with time to progression (TTP) greater than 6 months were categorised as responders, while those with TTP less than 6 months were classified as non‐responders.

In the biomarker discovery, candidate biomarkers were identified based on exo‐miRNAs that were differentially expressed between responders and non‐responders. To prioritise cancer tissue‐specific exo‐miRNA biomarkers, 1215 microRNAs (miRNAs) were selected—whose up‐ and down‐regulation between responders and non‐responders were matched in tissue‐ and exosome‐based profiling. Subsequently, among these, 344 miRNAs were also differentially expressed (|log_2_ fold change [FC]| > .5) in tissue‐based profiling (Figure 1A). Finally, eight exo‐miRNA candidates were prioritised that were significantly and differentially expressed (|log_2_ FC| > 2.0 and *p *< .01) between responders and non‐responders in exosome‐based profiling (Figure 1B,C). These eight exo‐miRNAs were identified as the potential predictor of resistance to chemotherapy in patients with mGC.

**FIGURE 1:**
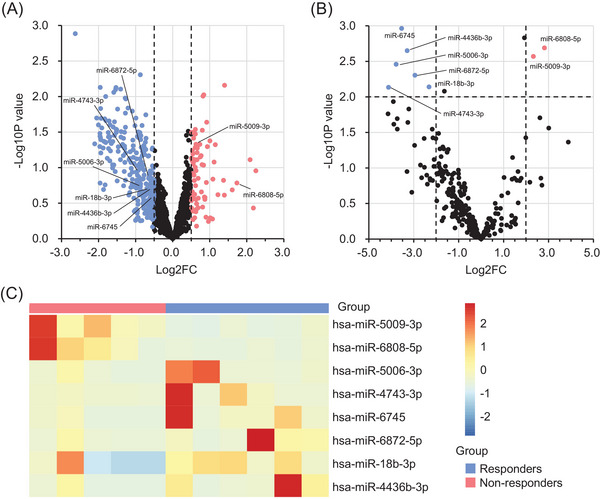
The biomarker discovery phase for the candidate exosomal microRNAs (exo‐miRNAs) to predict the response to systemic chemotherapy in patients with metastatic gastric cancer (mGC). (A) A volcano plot of microRNAs (miRNAs) for predicting the response to chemotherapy in tissue‐based small‐RNA sequence profiling. The red and blue dots represent up‐regulated (log_2_ fold change [FC] > .5) and down‐regulated (log_2_ FC < ‐.5) miRNAs in non‐responder patients, respectively. (B) A volcano plot of miRNAs for predicting the response to chemotherapy in exosome‐based small‐RNA sequence profiling. The red and blue dots represent significantly up‐regulated (log_2_ FC > 2.0 and *p *< .01) and down‐regulated (log_2_ FC < ‐2.0 and *p *< .01) in non‐responder patients, respectively. (C) Heatmap of eight candidate exo‐miRNAs in exosome‐based small‐RNA sequence profiling.

Next, using eight candidates, the exo‐miRNA panel was generated in qRT‐PCR assays using multivariate logistic regression analyses. In this biomarker training, our exo‐miRNA panel demonstrated a robust ability to predict non‐responders to chemotherapy, with an area under the curve (AUC) value of .83 (95% confidence interval [CI]: .63−1.00; Figure 2A,B). Of note, this exo‐miRNA panel demonstrated a more significant predictive potential compared to other clinicopathological signatures (Figure 2C). Furthermore, in the Kaplan–Meier analysis, the median PFS time was worse in patients with high score versus those with low score (.25 vs. .54 year; hazard ratio [HR]: 2.29; *p *< .05; Figure 2D). In multivariate logistic regression analyses, our exo‐miRNA panel was an independent predictor for lack of response in patients with mGC (odds ratio [OR]: 3.03; 95% CI: 1.06−8.66; *p *= .04; Table 1).

**FIGURE 2:**
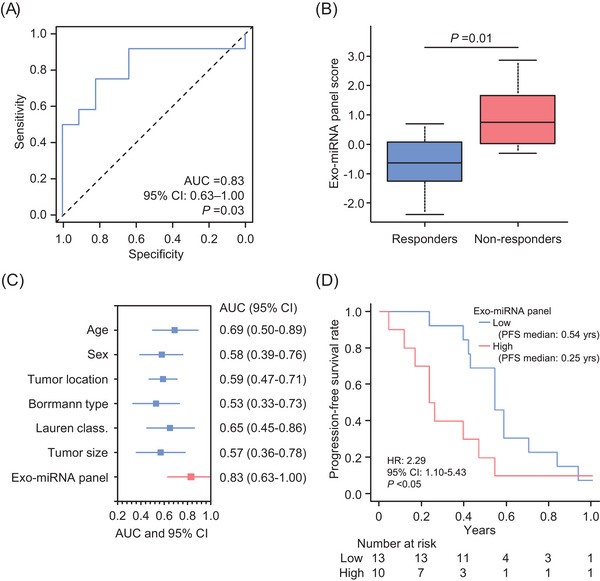
The clinical training phase of the exosomal microRNA (exo‐miRNA) panel for predicting the response to systemic chemotherapy in patients with metastatic gastric cancer (mGC). (A) Receiver operating characteristics (ROC) curve values for the exo‐miRNA panel in the clinical training cohort. (B) Box plots for the exo‐miRNA panel score in responder and non‐responder patients. (C) Forest plot with the area under the curve (AUC) values of key clinical characteristics and exo‐miRNA panel to predict the response to chemotherapy in patients with mGC. (D) Kaplan–Meier curves of the progression‐free survival for patients with exo‐miRNA panel high or low. CI, confidence interval; HR, hazard ratio.

**TABLE 1:** Univariate and multivariate analyses of key clinical factors predictive of response to chemotherapy in patients with metastatic gastric cancer.

	Univariate		Multivariate	
	OR (95% CI)	*p*‐Value	OR (95% CI)	*p*‐Value
Age, >59 years versus ≤59 years	.19 (.03–1.14)	.07	.12 (.02–1.26)	.08
Sex, female versus male	2.25 (.32–15.80)	.41		
Tumour location, upper versus middle and lower	1.33 (.56–3.16)	.99		
Borrmann type, type 4 versus others	1.33 (.22–7.98)	.75		
Lauren classification, diffuse versus intestinal	3.50 (.63–19.50)	.15		
Tumour size, >91 mm versus ≤91 mm	1.75 (.33–9.30)	.51		
exo‐miRNA panel score, high versus low	2.72 (1.07–6.91)	**.03**	3.03 (1.06–8.66)	**.04**

*Note*: Bold values denote statistical significance at the *p *< .05 level.

Abbreviations: CI, confidence interval; exo‐miRNA, exosomal microRNA; OR, odds ratio.

For the clinical biomarker validation, the qRT‐PCR assays were performed in an independent institution cohort. The exo‐miRNA panel revealed an impressive predictive potential for the resistance to chemotherapy, with a corresponding AUC value of .80 (95% CI: .64−.96; Figure 3A). Similar to the clinical training cohort, the predictive scores of non‐responders were significantly higher than those of responders (*p *= .03; Figure 3B), and the median PFS time was shorter in patients with high score versus those with low score (.25 vs. .50 year; HR: 1.95; *p *< .05; Figure 3C). Interestingly, when we analysed the subset of patients with human epidermal growth factor receptor type 2 (HER2)‐negative and mismatch repair (MMR)‐proficient tumours, who tend not to benefit from targeted therapy and immunotherapy, our panel accurately predicted non‐responders, with an AUC value of .85 and .81, respectively (Figure 3D). Overall, the robust exo‐miRNA‐based liquid biopsy panel was evaluated by a comprehensive genome wide biomarker discovery, followed by qRT‐PCR‐based biomarker training and validation.

**FIGURE 3:**
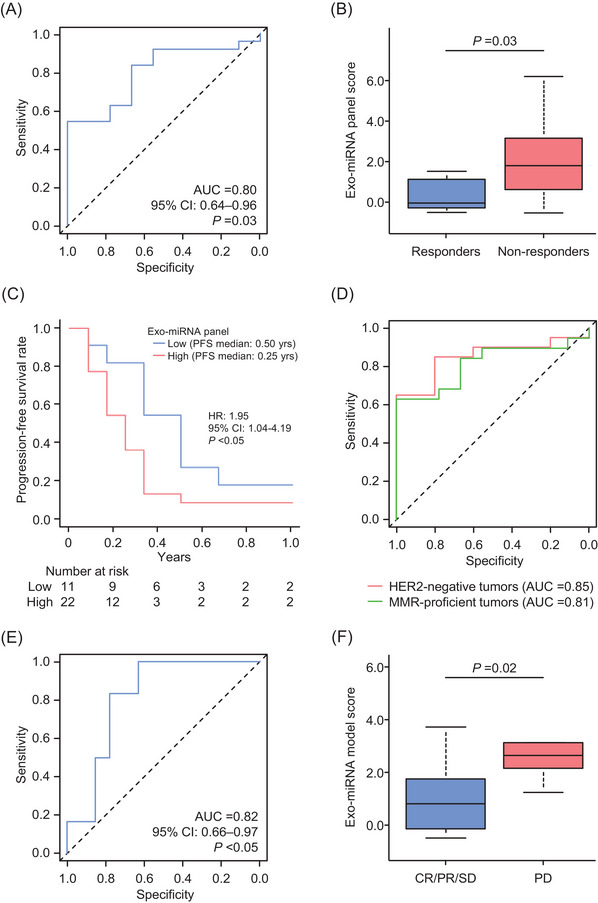
Clinical validation phase for the exosomal microRNA (exo‐miRNA) panel in predicting the response to systemic chemotherapy in patients with metastatic gastric cancer (mGC). (A) Receiver operating characteristics (ROC) curve values for the exo‐miRNA panel in the clinical validation cohort. (B) Box plots for the exo‐miRNA panel score in responder and non‐responder patients. (C) Kaplan–Meier curves of the progression‐free survival for patients with exo‐miRNA panel high or low. (D) ROC curve values for the exo‐miRNA panel in patients with human epidermal growth factor receptor type 2 (HER2)‐negative and mismatch repair (MMR)‐proficient tumours. (E) ROC curve values for exo‐miRNA model to predict progressive disease (PD) in the Response Evaluation Criteria in Solid Tumours (RECIST) guideline in the clinical validation cohort. (F) Box plots for the exo‐miRNA model score in PD and other response groups. AUC, area under the curve; CI, confidence interval; CR, complete response; HR, hazard ratio; PR, partial response; SD, stable disease.

For further confirmation of the exo‐miRNA model's predictive potential, additional analyses were performed to determine whether our model could predict the Response Evaluation Criteria in Solid Tumours (RECIST) guideline‐based response in the clinical validation cohort.^8^ It was reassuring to note that our model accurately predicted therapeutic response in patients whose best response was progressive disease, with an AUC value of .82 (95% CI: a.66−.97; Figure 3E,F). Collectively, our liquid biopsy model robustly predicted even the RECIST guideline‐based response, highlighting its potential predictive efficacy and versatility in clinical practice.

To clarify the functional relevance of our miRNA biomarkers, the miRNA target gene analyses were performed using the miRDB database,^9^ and a total of 1247 genes were detected as target genes of each miRNA (target score > 80). The enrichment pathway analyses using the DAVID bioinformatic database^10^ revealed that these target genes were enriched (fold enrichment > 2.0 and *p* < .05) in multiple cancer‐related signalling pathways, such as Hedgehog signalling pathway, transforming growth factor (TGF)‐beta signalling pathway and mitogen‐activated protein kinase (MAPK) signalling pathway (Figure S2), highlighting our biomarkers’ functional relevance in the response of chemotherapy in patients with mGC.

We would like to acknowledge some of the limitations of our study. First, the clinical biospecimens in our study were retrospectively collected with relatively modest size patient cohorts. In the future, a well‐designed prospective study must be needed to confirm this model further. Second, the patient cohorts in this study were of Asian heritage. The disease aetiology of GC varies between East Asia and Western countries; therefore, to ensure the generalisability of our assay, our findings need to be validated in cohorts from other patient populations. Despite these limitations, our robust exo‐miRNA‐based liquid biopsy model could help the development of precision medicine for patients with mGC.

In conclusion, we successfully established an exo‐miRNA‐based liquid biopsy assay that allows the robust, accurate and less‐invasive prediction of resistance to first‐line standard systemic chemotherapy in patients with mGC. These findings can help categorise patients for more effective therapeutic approaches in pre‐treatment settings for patients with mGC.

## AUTHOR CONTRIBUTIONS

*Study concept and design*: Keisuke Okuno, Masanori Tokunaga, Minoru Tanabe, Yusuke Kinugasa and Ajay Goel. *Provision of samples*: Hoon Hur, Jeeyun Lee and Joon Oh Park. *Acquisition of clinical data*: Keisuke Okuno, Hoon Hur, Jeeyun Lee, Joon Oh Park and Ajay Goel. *Analysis and interpretation of data*: Keisuke Okuno, Shuichi Watanabe and Ajay Goel. *Statistical analysis*: Keisuke Okuno and Shuichi Watanabe. *Drafting of the manuscript*: Keisuke Okuno, Shuichi Watanabe, Hoon Hur, Jeeyun Lee, Joon Oh Park, Masanori Tokunaga, Minoru Tanabe, Yusuke Kinugasa and Ajay Goel.

## CONFLICT OF INTEREST STATEMENT

The authors declare they have no conflicts of interest.

## ETHICS STATEMENT

A written informed consent was obtained from all patients for their willingness to participate in this study, and the study was performed as per Helsinki declarations following approval by the institutional review boards of all participating institutions.

## CONSENT FOR PUBLICATION

Not applicable. The manuscript does not contain any individual personal data.

## Supporting information

Supporting Information

## Data Availability

The datasets generated and analysed during the current study are available from the corresponding author upon reasonable request.

## References

[1] Ajani JA , D'Amico TA , Bentrem DJ , et al. Gastric cancer, version 2.2022, NCCN clinical practice guidelines in oncology. J Natl Compr Canc Netw. 2022;20(2):167‐192. doi:10.6004/jnccn.2022.0008 35130500

[2] Japanese gastric cancer treatment guidelines 2018 (5th edition). Gastric Cancer. 2021;24(1):1‐21. doi:10.1007/s10120-020-01042-y 32060757 PMC7790804

[3] Koizumi W , Narahara H , Hara T , et al. S‐1 plus cisplatin versus S‐1 alone for first‐line treatment of advanced gastric cancer (SPIRITS trial): a phase III trial. Lancet Oncol. 2008;9(3):215‐221. doi:10.1016/s1470-2045(08)70035-4 18282805

[4] Cunningham D , Starling N , Rao S , et al. Capecitabine and oxaliplatin for advanced esophagogastric cancer. N Engl J Med. 2008;358(1):36‐46. doi:10.1056/NEJMoa073149 18172173

[5] Yamada Y , Higuchi K , Nishikawa K , et al. Phase III study comparing oxaliplatin plus S‐1 with cisplatin plus S‐1 in chemotherapy‐naïve patients with advanced gastric cancer. Ann Oncol. 2015;26(1):141‐148. doi:10.1093/annonc/mdu472 25316259

[6] Shah MA , Bang YJ , Lordick F , et al. Effect of fluorouracil, leucovorin, and oxaliplatin with or without onartuzumab in HER2‐negative, MET‐positive gastroesophageal adenocarcinoma: the METGastric randomized clinical trial. JAMA Oncol. 2017;3(5):620‐627. doi:10.1001/jamaoncol.2016.5580 27918764 PMC5824210

[7] Van Cutsem E , Moiseyenko VM , Tjulandin S , et al. Phase III study of docetaxel and cisplatin plus fluorouracil compared with cisplatin and fluorouracil as first‐line therapy for advanced gastric cancer: a report of the V325 Study Group. J Clin Oncol. 2006;24(31):4991‐4997. doi:10.1200/jco.2006.06.8429 17075117

[8] Eisenhauer EA , Therasse P , Bogaerts J , et al. New response evaluation criteria in solid tumours: revised RECIST guideline (version 1.1). Eur J Cancer. 2009;45(2):228‐247. doi:10.1016/j.ejca.2008.10.026 19097774

[9] Chen Y , Wang X . miRDB: an online database for prediction of functional microRNA targets. Nucleic Acids Res. 2020;48(D1):D127‐D131. doi:10.1093/nar/gkz757 31504780 PMC6943051

[10] Sherman BT , Hao M , Qiu J , et al. DAVID: a web server for functional enrichment analysis and functional annotation of gene lists (2021 update). Nucleic Acids Res. 2022;50(W1):W216‐W221. doi:10.1093/nar/gkac194 35325185 PMC9252805

