# Supplementary material for: An exosome‐based liquid biopsy signature for therapeutic response prediction in metastatic gastric cancer
Source: Clin Transl Med. 2024 Jul 19;14(7):e1629. doi: 10.1002/ctm2.1629 (PMC11259598; doi:10.1002/ctm2.1629)
Supplement: Supplementary file 1 — Supporting Information [file CTM2-14-e1629-s001.docx]

**Supplementary Figures**

**Supplementary Figure S1: The schematic of the study design.** GC, gastric cancer; FC, fold change; miRNA, microRNA; qRT-PCR, quantitative reverse transcription polymerase chain reaction; AUC, area under the curve.


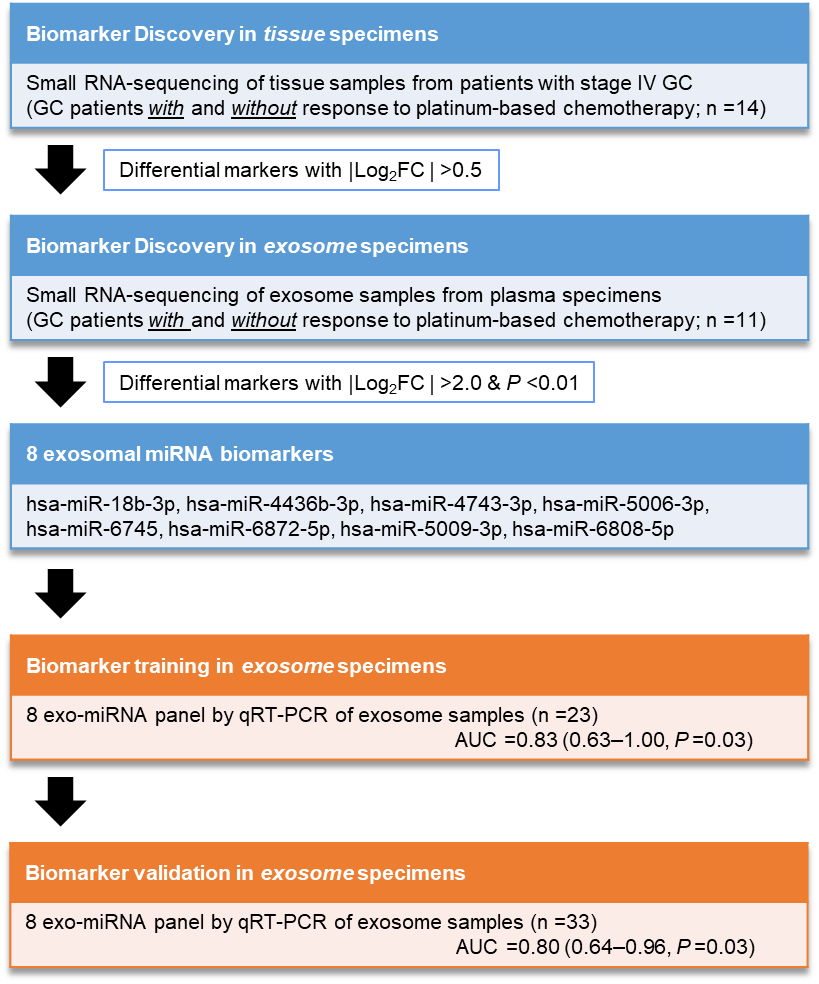


**Supplementary Figure S2: Scatter plots of KEGG pathway enrichment analysis of target genes of candidate miRNA biomarkers.** The circle area indicates the number of target genes in the pathway, and the circle color represents the range of the *P* value. miRNA, microRNA.


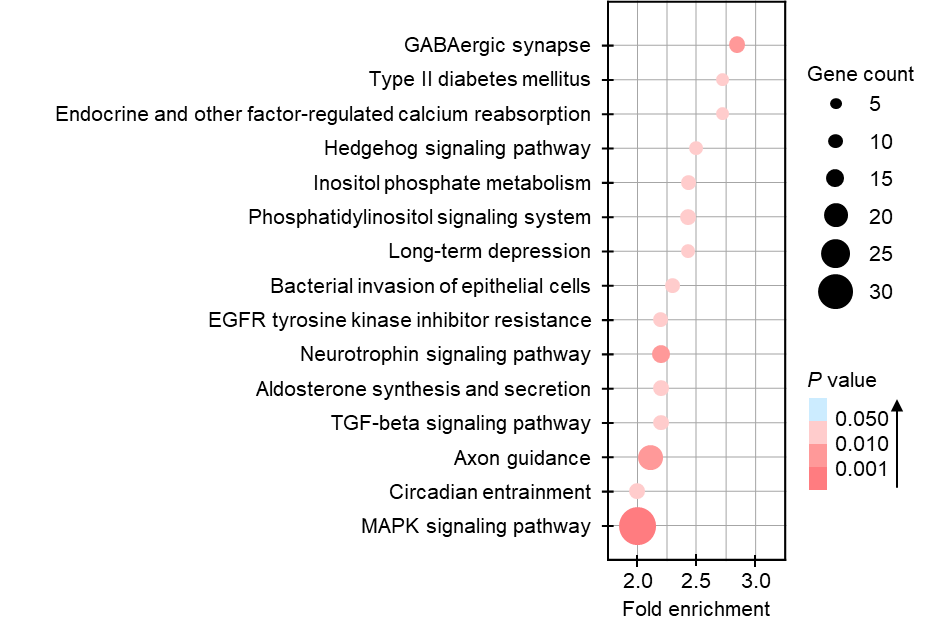


**Supplementary Tables**

**Supplementary Table S1: Patient characteristics of the biomarker discovery cohort**

|  | | | | | | |
| --- | --- | --- | --- | --- | --- | --- |
|  | **Tissue**  **-based discovery** (n =14) | | | **Exosome-based discovery** (n =11) | | |
|  | n |  | (%) | n |  | (%) |
| Age, mean (±SD) (years) | 65 |  | (±11) | 63 |  | (±14) |
| Sex |  |  |  |  |  |  |
| Male | 11 |  | (79) | 9 |  | (82) |
| Female | 3 |  | (21) | 2 |  | (18) |
| Tumor location |  |  |  |  |  |  |
| Upper third | 1 |  | (7) | 2 |  | (18) |
| Middle third | 5 |  | (36) | 3 |  | (27) |
| Lower third | 8 |  | (57) | 6 |  | (55) |
| Borrmann type |  |  |  |  |  |  |
| Type 2 | 3 |  | (21) | 2 |  | (18) |
| Type 3 | 6 |  | (43) | 6 |  | (55) |
| Type 4 | 5 |  | (36) | 3 |  | (27) |
| Tumor size, mean (±SD) (cm) | 7.6 |  | (±2.6) | 8.6 |  | (±3.3) |
| Lauren classification |  |  |  |  |  |  |
| Intestinal | 4 |  | (29) | 2 |  | (18) |
| Diffuse | 10 |  | (71) | 9 |  | (82) |
| Responder |  |  |  |  |  |  |
| Responder | 7 |  | (50) | 6 |  | (55) |
| Non-responder | 7 |  | (50) | 5 |  | (45) |
| SD, standard deviation | | | | | | |

**Supplementary Table S2: Patient characteristics of the clinical training cohort**

|  | | | |
| --- | --- | --- | --- |
|  | **Clinical training** (n =23) | | |
|  | n |  | (%) |
| Age, mean (±SD) (years) | 59 |  | (±12) |
| Sex |  |  |  |
| Male | 17 |  | (74) |
| Female | 6 |  | (26) |
| Tumor location |  |  |  |
| Upper third | 2 |  | (9) |
| Middle third | 12 |  | (52) |
| Lower third | 9 |  | (39) |
| Borrmann type |  |  |  |
| Type 2 | 6 |  | (26) |
| Type 3 | 10 |  | (43) |
| Type 4 | 7 |  | (31) |
| Tumor size, mean (±SD) (cm) | 9.1 |  | (±3.5) |
| Lauren classification |  |  |  |
| Intestinal | 11 |  | (48) |
| Diffuse | 12 |  | (52) |
| 1st line chemotherapy regimen |  |  |  |
| SP | 4 |  | (17) |
| FOLFOX | 12 |  | (52) |
| CAPOX | 2 |  | (9) |
| Others | 5 |  | (22) |
| Responder |  |  |  |
| Responder | 11 |  | (48) |
| Non-responder | 12 |  | (52) |
| SD, standard deviation; SP, S-1 + Cisplatin; FOLFOX, Fluorouracil + Oxaliplatin + Leucovorin; CAPOX, Capecitabine + Oxaliplatin | | | |

**Supplementary Table S3: Patient characteristics of the clinical validation cohort**

|  | | | |
| --- | --- | --- | --- |
|  | **Clinical validation** (n =33) | | |
|  | n |  | (%) |
| Sex |  |  |  |
| Male | 19 |  | (58) |
| Female | 14 |  | (42) |
| HER2 |  |  |  |
| Negative | 25 |  | (76) |
| Positive | 2 |  | (6) |
| Unknown | 6 |  | (18) |
| MMR |  |  |  |
| Proficient | 28 |  | (85) |
| Deficient | 2 |  | (6) |
| Unknown | 3 |  | (9) |
| 1st line chemotherapy regimen |  |  |  |
| SP | 2 |  | (6) |
| CAPOX | 31 |  | (94) |
| RECIST version 1.1 |  |  |  |
| CR/PR | 17 |  | (52) |
| SD | 10 |  | (30) |
| PD | 6 |  | (18) |
| Responder |  |  |  |
| Responder | 9 |  | (27) |
| Non-responder | 24 |  | (73) |
| SP, S-1 + Cisplatin; CAPOX, Capecitabine + Oxaliplatin; HER2, human epidermal growth factor receptor 2; MMR, mismatch repair; RECIST, response evaluation criteria in solid tumors; CR, complete response; PR, partial response; SD, stable disease; PD, progressive disease | | | |
